# Non-invasive physiological assessment of coronary artery obstruction on coronary computed tomography angiography

**DOI:** 10.1007/s12471-024-01902-7

**Published:** 2024-10-07

**Authors:** Leonie M. Becker, Joyce Peper, Sophie H. van Nes, Hendrik W. van Es, Krischan D. Sjauw, Tim P. van de Hoef, Tim Leiner, Martin J. Swaans

**Affiliations:** 1 Department of Cardiology, St. Antonius Hospital, Nieuwegein, The Netherlands; 2 Department of Radiology, University Medical Centre Utrecht, Utrecht, The Netherlands; 3 Department of Radiology, St. Antonius Hospital, Nieuwegein, The Netherlands; 4 Department of Cardiology, University Medical Centre Utrecht, Utrecht, The Netherlands; 5 Department of Radiology, Mayo Clinics, Rochester, MN USA

**Keywords:** Computed tomography angiography, Coronary artery disease, CT-derived fractional flow reserve, Myocardial ischaemia, Artificial intelligence

## Abstract

Computed tomography-derived fractional flow reserve (CT-FFR) enhances the specificity of coronary computed tomography angiography (CCTA) to that of the most specific non-invasive imaging techniques, while maintaining high sensitivity in stable coronary artery disease (CAD). As gatekeeper for invasive coronary angiography (ICA), use of CT-FFR results in a significant reduction of negative ICA procedures and associated costs and complications, without increasing cardiovascular events. It is expected that CT-FFR algorithms will continue to improve, regarding accuracy and generalisability, and that introduction of new features will allow further treatment guidance and reduced invasive diagnostic testing. Advancements in CCTA quality and artificial intelligence (AI) are starting to unfold the incremental diagnostic and prognostic capabilities of CCTA’s attenuation-based images in CAD, with future perspectives promising additional CCTA parameters which will enable non-invasive assessment of myocardial ischaemia as well as CAD activity and future cardiovascular risk. This review discusses practical application, interpretation and impact of CT-FFR on patient care, and how this ties into the CCTA ‘one stop shop’ for coronary assessment and patient prognosis. In this light, selective adoption of the most promising, objective and reproducible techniques and algorithms will yield maximal diagnostic value of CCTA without overcomplicating patient management and guideline recommendations.

## Introduction

Invasive coronary angiography (ICA) with invasive pressure measurements, such as fractional flow reserve (FFR), instantaneous wave-free ratio (iFR) and resting full-cycle ratio (RFR), is the current diagnostic standard for ischaemia-inducing epicardial coronary artery disease (CAD) [1]. As the majority of patients with anginal symptoms do not have obstructive epicardial CAD, routine ICA with pressure measurements is undesirable due to the invasive nature and associated risks and costs [2, 3]. Consequently, current international guidelines recommend functional or anatomical non-invasive imaging modalities for initial screening [1].

Functional imaging modalities, including single-photon emission computed tomography (SPECT), positron emission tomography (PET), magnetic resonance imaging (MRI) and stress echocardiography, detect (inducible) ischaemia. They are generally more specific but less sensitive than anatomical tests and do not capture coronary anatomy or plaque burden, which provide important prognostic information [4, 5]. Coronary computed tomography angiography (CCTA) is used as an anatomical screening tool for CAD. Advantages include low costs, quick acquisition protocol and excellent negative predictive value. However, specificity is lacking as stenosis severity is easily overestimated, especially in calcified lesions, and more than half of the patients with stenoses between 50–90% do not have ischaemia [1–3, 6]. Functional testing is recommended in these cases, which still results in a substantial proportion of negative ICA procedures [1, 2].

Recent advancements in computational fluid dynamics (CFD) and artificial intelligence (AI) have revolutionised non-invasive detection of ischaemia using CCTA data [7, 8]. Computed tomography-derived fractional flow reserve (CT-FFR) enhances CCTA specificity to levels comparable with the most specific non-invasive imaging techniques in patients with stable CAD, while maintaining high sensitivity, resulting in a significant reduction in negative ICA procedures and associated costs and complications [4]. CT-FFR provides a non-invasive estimation of FFR without the need for arterial puncture, additional radiation, contrast medium or medication, and requires no changes in CCTA acquisition protocols [3]. Additionally, analysis results are generally available before the next outpatient consultation, maximising certainty regarding the results for both patients and treating physicians.

**Fig. 1:**
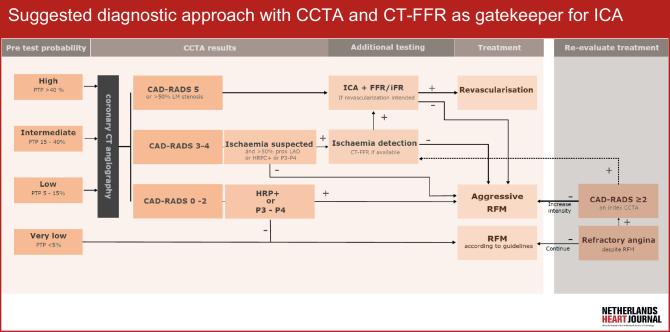
Infographic: Suggested diagnostic approach using CCTA and CT-FFR as gatekeepers for ICA in clinical management and cardiovascular risk modification. *PTP* pre-test probability, *CAD-RADS* coronary artery disease—reporting and data system, *LM* left main, *LAD* left anterior descending, *HRP* high-risk plaque, *P3–P4* plaque burden severe-extensive, *ICA* invasive coronary angiography, *FFR* fractional flow reserve, *iFR* instantaneous wave-free ratio, *CT-FFR* computed tomography-derived fractional flow reserve, *RFM* risk factor modification

This review discusses the technical principles and interpretation of CT-FFR, impact of its integration into routine patient care (Fig. 1), and how this ties into the CCTA ‘one stop shop’ for coronary assessment and patient prognosis.

## CT-FFR computation

The process of deriving CT-FFR from CCTA involves several steps. Firstly, reconstruction of a detailed, three-dimensional model of the coronary tree from the CCTA image data. After this, CFD-based CT-FFR modalities calculate the inflow and outflow boundary conditions and apply the required assumptions to simulate hyperaemia. Subsequently, coronary flow, pressure and resistance are simulated by solving CFD equations along the entire vessel, after which FFR values are calculated [3, 9]. AI approaches, encompassing machine learning, deep learning and radiomics, have been employed for CT-FFR as well [7].

### 3D-coronary tree reconstruction

Accurate automated coronary artery segmentation from CCTA data is affected by image artifacts, similar CT characteristics of surrounding structures, small coronary diameters and differences in coronary anatomy, plaque composition and plague burden between individuals [10, 11]. Pre-processing with vessel feature enhancement methods addresses these issues by artifact reduction and identification of vascular structures, improving their contrast while suppressing non-vascular structures [10]. Anisotropic diffusion filters, Hessian-based vessel enhancement, especially Frangi’s filter, and multi-planar reformation are the most used methods [10]. Vessel feature enhancement adds significant computing time and complexity to the segmentation process, especially compared with the limited processing time required for deep learning-based segmentation. Therefore, it is no longer present in newer methods [10].

Segmentation methods can be divided into various categories and subcategories. Initially, region growing methods were mostly used, of which active contouring was the most popular. These add, in a stepwise fashion, neighbouring voxels with similar image features into regions representing structural classes [10].

Over the years, deep-learning based segmentation has gained prominence, in which convolutional neural networks (CNN) develop the applied filters independently during training. Voxelwise probability maps construct a 3D coronary model by assessing probabilities of voxels being part of the coronary tree using imaging features [10]. Global partitioning methods work by grouping data into regions with similar properties. For example, in graph cut-based segmentation voxels are sorted in a graph, which is subsequently divided into foreground and background sub-graphs [10].

### Computational fluid dynamics-based algorithms

For CFD analysis, the generated 3D lumen is divided into a mesh containing hundreds of thousands of volumetric elements [12]. Subsequently, a mathematical coronary physiology model calculates inflow and outflow boundary conditions. The key assumptions for this are derived from physiological principles, such as Murray’s law and form-function relationships, and vary per algorithm [13]. For HeartFlow FFR-CT, the first commercially available CT-FFR algorithm, these are: 1) coronary blood flow at rest is proportionate to the myocardial mass, which can be derived from CCTA images, 2) vascular resistance is inversely proportionate to lumen diameter and 3) microvascular resistance during hyperaemia is predictable. In this software, hyperaemic microvascular resistance is set at 24% of baseline resistance for the calculations [15].

Coronary inflow is derived from aortic outflow, which can be calculated as steady-state outflow, with the stroke volume derived from the difference between end-diastolic and end-systolic volume, or as pulsatile flow, using a continuous flow waveform [16]. Although the correlation of CT-FFR values between the simpler steady-state outflow and pulsatile outflow appeared good in a small comparative study, pulsatile flow is more patient-specific and steady-state models might therefore be less accurate [16, 17]. For the outflow boundary, baseline microvascular resistance can be modelled in two- or three-element Windkessel models, or as an outflow outlet condition with flowrate specified at each outlet [17].

For CFD-based CT-FFR calculations, blood is generally treated as an incompressible Newtonian fluid with pre-specified density and viscosity. To calculate coronary flow and pressure, Navier-Stokes equations, the partial differential equations that govern fluid dynamics, are solved for each volumetric element at thousands of timepoints in the cardiac cycle [13].

The number of volumetric elements and the complexity of applied assumptions and boundary conditions affect the computational power required to run the analysis. Simpler CT-FFR algorithms, such as reduced-order and steady-state models, require less power, allowing for on-site CT-FFR calculation [12, 18]. However, they are less reliable when computing more complicated anatomical and lesion characteristics, such as small vessels, side branches, bifurcation areas and eccentric stenoses [19].

HeartFlow Inc. (Redwood City, California, USA) developed the first commercially available CT-FFR software. Their offsite algorithm is powered by AI and supervised and corrected by trained analysts. In extensive validation against FFR in patients with stable CAD, HeartFlow FFR-CT showed high sensitivity and specificity (78% and 80%, respectively) [20]. Studies like DISCOVER-FLOW, DEFACTO and NXT demonstrated enhanced diagnostic accuracy for haemodynamically significant CAD with HeartFlow FFR-CT compared with CCTA alone [8, 9, 21]. The PACIFIC CT-FFR sub-analysis reported the highest diagnostic performance for CT-FFR at vessel level compared with SPECT, PET and CCTA, although PET outperformed CT-FFR in the intention-to-diagnose analysis due to high CCTA rejection rate [4].

Other CFD-based CT-FFR models, such Siemens cFFR, Philips IntelliSpace CT-FFR, Canon Medical Systems CT-FFR, ANSYS FLUENT, COMSOL multiphysics and PowerFlow, are not yet externally validated and exclusively accessible for research purposes [20]. A meta-analysis including multiple CFD-based CT-FFR algorithms indicated comparable diagnostic performance (sensitivity, specificity, and accuracy) between them [20].

### AI-based CT-FFR models

AI models extract characteristics from a derivation cohort to establish their algorithm [22]. AI CT-FFR can be based on machine learning, deep learning or radiomics. In machine learning models, analysable characteristics are inserted manually. In deep learning models, CNN automatically detect characteristics relevant for the task at hand. This requires larger amounts of training data, but potentially results in higher accuracy due to identification of characteristics undiscernible to humans. CNN training can be supervised, semi-supervised or unsupervised. Radiomics is quantitative advanced feature analysis to extract large amounts of mathematical characteristics from images [23].

General advantages of AI-based models include low processing times, in-house availability, capability to identify and assess complex relationships and large datasets, elimination of human errors and fatigue, and potential for self-improvement [10, 23]. On the other hand, the training method of AI-based models carries an inherent risk of underfitting or overfitting to the training data, which can be hard to detect and leads to loss of generalisability [23]. Large amounts of good quality data, meaning accurately labelled, representative of the target population and including reference standards, is paramount when training AI models, and extensive (external) validation using different datasets of equal high quality and representativeness is of crucial importance before these models can be deployed in routine clinical care [23]. To improve individual specificity, some AI models incorporate patient factors such as age, gender, blood pressure and clinical data into their FFR estimation [24]. Additionally, as good quality CCTA datasets for training and validation are rare, some developers include synthetic coronary tree models in their algorithm training [24]. Several studies have been published using AI-based models such as SPIMED-AI CorEx, DeepVESSEL-FFR, uCT-FFR, Beijing Heartcentury and AccuFFRCT [25, 26]. Currently, only DeepVESSEL-FFR (KeyaMedical) is FDA approved.

AI algorithms for CCTA assessment have been developed as well, with AI-aided atherosclerosis imaging and quantitative cardiac CT (AI-QCT, Cleerly Inc.) being the most known. AI-QCT is an FDA-approved, commercially available algorithm developed for quick and accurate automated CCTA assessment. The algorithm generates a 3D model of the coronary arteries, identifies stenoses and quantifies plaque. It reports the CAD burden as a level between ‘none’ and ‘severe’ with localisation and composition of individual plaques. Application of AI-QCT in the CONSERVE trial showed a potential reduction in unnecessary ICA of approximately 90% [27].

### Studies on outcome and clinical management

Various studies and trials have explored outcomes and clinical management associated with CT-FFR in stable CAD.

Most studies on outcomes used HeartFlow FFR-CT. The PLATFORM study compared CCTA with CT-FFR to standard of care in patients designated for ICA. Care guided by CTA and selective CT-FFR yielded equivalent clinical outcomes and quality of life, and lower costs over 1 year [28]. Additionally, only 12% of ICA procedures following a CCTA/CT-FFR guided strategy revealed no obstructive CAD, compared with 73% of ICA procedures in the primary invasive strategy [28]. In the randomised FORECAST trial, involving 1400 patients, CTCA with CT-FFR reduced ICA compared with standard clinical care without significant difference in costs [29]. In the PROMISE trial, involving 2103 patients, deferred testing in minimal-risk patients and CCTA with CT-FFR in the rest led to reduction of ICA without obstructive CAD, without a statistically significant impact on death and nonfatal myocardial infarction at 1 year [30]. This ‘precision strategy’ was therefore considered a safe and efficient diagnostic approach for patients suspected of stable CAD. Registries like ADVANCE, RIPCORD and IMPACT-FFR showed significant reduction in (invasive) diagnostic testing, increased efficiency and good prognostic value for CT-FFR. CT-FFR might also improve outcomes, particularly due to identification of higher-risk patients [3, 31, 32]. In the TARGET trial, using the DEEPVESSEL-FFR algorithm, CT-FFR guidance reduced unnecessary invasive procedures, and demonstrated a trend toward lower costs without affecting cardiovascular events at 1 year [7].

Overall, these findings emphasise the potential benefits of CCTA and CT-FFR guidance in stable CAD: improved efficiency, risk stratification and outcomes, and cost-effectiveness. However, widespread use is hindered, particularly by cost and reimbursement limitations. In the Netherlands, the randomised iCORONARY [33] and FUSION [34] trials are underway, comparing CT-FFR (both) and ICA-based quantitative flow ratio (QFR, iCORONARY) with standard of care: ICA with FFR, in stable CAD. These studies are designed to assess safety, unnecessary ICA, cost-effectiveness and quality of life. Successful completion is expected to result in CT-FFR reimbursement and routine availability.

### CT-FFR interpretation and reliability

Generally, CT-FFR results are presented in a colour-coded 3D model containing FFR values for every point of the coronary tree. Similar to FFR, CT-FFR values > 0.80 are considered normal, meaning ICA can be safely deferred in favour of medical therapy [15]. Values ≤ 0.75 indicate ischaemia, and values of 0.76–0.80 are considered borderline. Ischaemia is present in approximately half of the patients with borderline CT-FFR values. A meta-analysis by Celeng et al., which only contained CFD-based CT-FFR algorithms, revealed a sensitivity of ≥ 90% at CT-FFR values > 0.82 and specificity ≥ 90% at CT-FFR values ≤ 0.74, while CT-FFR diagnostic accuracy dropped from 87 to 54% in the zone between 0.74 and 0.82. [20] A recent meta-analysis by Faulder et al., including multiple AI-based CT-FFR algorithms, reported a grey zone between 0.64 and 0.85 [25].

**Fig. 2:**
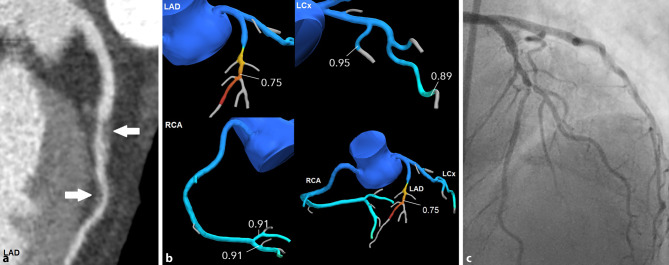
Example of a patient with **a** an intermediate coronary stenosis in the left anterior descending artery (LAD) with **b** corresponding CT-FFR analysis positive for ischaemia and **c** subsequent invasive coronary angiography, during which revascularisation of both focal stenoses was performed. *RCA* right coronary artery, *LCx* left circumflex artery

**Fig. 3:**
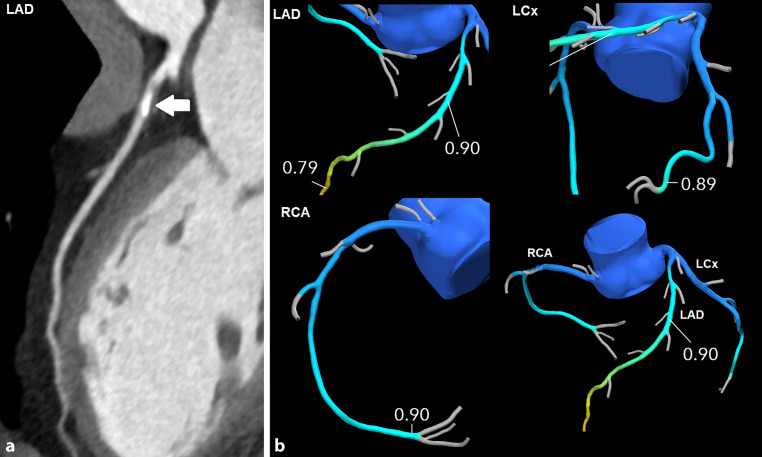
Example of a patient with **a** an intermediate coronary stenosis in the left anterior descending artery (LAD) with **b** corresponding CT-FFR analysis negative for ischaemia. No invasive coronary angiography was performed. Please note that the CT-FFR value decreases along all three coronary vessels, despite absence of other focal stenoses

Studies have shown that CT-FFR values are most representative 1–2 cm distal to a focal lesion (Figs. 2 and 3; [19]). CT-FFR stenosis overestimation is the most frequently occurring discordance with FFR, as false-negatives are low [8, 9, 21, 35]. In the early CT-FFR studies, interpretation was based on the lowest CT-FFR value in each coronary artery, usually the most distal value. This resulted in increased overestimation of stenoses, as CT-FFR values have a tendency to decrease from proximal to distal (Figs. 2 and 3) and may even indicate distal ischaemia in the absence of focal stenoses, especially in diffuse coronary artery disease or small vessels [15].

Motion artifacts are the most important cause of CCTA rejection for CT-FFR [19]. A heart rate < 60 beats per minute at acquisition is therefore strongly recommended [8, 9]. Other factors that influence CT-FFR through image quality are image noise and artifacts, for example caused by prosthetic heart valves, pacemakers, internal defibrillators, leads and heavy plaque calcification [18]. Bifurcation lesions and CCTA acquisition without nitroglycerin can affect CT-FFR reliability [18]. Generally, CT-FFR algorithms are not validated in patients with previous revascularisation, acute ischaemia, severe aortic valve stenosis, complex congenital heart diseases, haemodynamic instability or BMI > 35 [18].

## Considerations in decision-making

Gatekeeping CCTA acquisition with calcium score thresholds often leads to unnecessary invasive assessment of CAD burden, while the most vulnerable plaques remain undetected, as calcification is a sign of plaque stabilisation [36]. Therefore, symptomatic patients with pre-test probability of CAD > 5% should be considered for CCTA [36, 37]. Regardless of the result of CCTA, cardiovascular risk modification and, if symptomatic, anti-anginal medication are indicated in all patients. This should be intensified in patients with high plaque burden, who are at higher risk of events [1, 5]. The Coronary Artery Disease Reporting and Data System (CAD-RADS) score was recently updated to incorporate overall coronary plaque burden (P1–P4), presence of high-risk plaque (HRP) characteristics and CT-FFR or CT-perfusion results (I- or I+) [5].

In patients with CAD-RADS 0–2 (< 50% stenosis) on CCTA, ischaemia is rare [1, 5, 36]. For CAD-RADS 3–5, symptoms, lesion localisation and characteristics need to be taken into account for clinical management [5]. Left main and proximal left anterior descending artery stenoses are considered high risk, and HRP increase the chance of lesion ischaemia [1, 5]. Ischaemia detection is advised in these patients if they are symptomatic [1, 5, 38]. In other cases, ischaemia detection can be deferred until refractory angina becomes apparent, as cardiovascular risk modification and anti-anginal medication result in satisfactory reduction of angina burden in most patients with obstructive CAD on CCTA without an increase in cardiovascular events [14, 38, 39]. The randomised CLEAR-CAD trial aims to assess the cost-effectiveness and quality of life associated with this strategy compared with standard of care. In the CLEAR-CAD trial, all patients receive CCTA with appropriate optimal medical therapy, non-invasive ischaemia detection is reserved for those with refractory angina, and ICA and revascularisation are only performed in case of refractory angina and substantial ischaemia [40].

CT-FFR is a good option for non-invasive ischaemia detection in patients with CAD-RADS 3–4, and can be considered to rule-out ischaemia in patients with proximal CAD-RADS 2 lesions and refractory angina [9]. As lesions > 90% generally cause ischaemia, functional tests such as CT-FFR are theoretically unnecessary, although clinicians need to consider the possibility of stenosis overestimation on CCTA [1, 15]. ICA with invasive pressure measurements or coronary physiology can be considered for CAD-RADS 4–5, in patients with positive or borderline CT-FFR results and in patients with CT-FFR values < 0.85 and refractory angina [1, 20, 25, 29]. It is recommended that lesion location, patient- and symptom characteristics are taken into consideration with CT-FFR values for clinical management [19].

The infographic graphically represents the above outlined suggested integration of CT-FFR in clinical management.

## Future perspectives

CCTA’s attenuation-based images hold incremental physiological details which could significantly enhance diagnostic specificity and facilitate more personalised treatment and prognosis. A promising CCTA parameter is the fat attenuation index (FAI), which represents the degree of coronary inflammation. Recently, low-dose anti-inflammatory colchicine was approved by the FDA for cardiovascular risk modification, based on studies on CAD and elevated serum high-sensitive C‑reactive protein. The FAI captures coronary-specific inflammation, aiding identification of individuals at risk, but also assesses risk modification effectiveness on disease activity [41, 42]. Another promising parameter was reported by Bom et al. In their study, CT-FFR showed only slightly higher diagnostic accuracy for haemodynamically obstructive CAD than dividing subtended myocardial mass by the minimal lumen area squared (Vsub/MLA^2^) [43]. Additional promising CCTA parameters are epicardial adipose tissue volume and distribution, plaque characteristics in other vessels and left ventricular mass [44–46].

In photon-counting CT, the energy signals of X‑ray photons are registered individually. This leads to significant improvement in CT resolution with reduction of noise, artifacts and radiation dose [47, 48]. For CCTA, this increases accuracy of stenosis severity, plaque burden and HRP assessment, and reduces artifacts. This subsequently improves the accuracy of software based on CCTA images, such as CT-FFR.

It is expected that CT-FFR and AI algorithms themselves will also continue to improve, regarding accuracy as well as generalisability, and new features will further guide treatment and reduce invasive diagnostic testing. Examples are simulated PCI results, non-invasive functional SYNTAX score for Heart Team discussions, calculation of percentage myocardium at risk, individual assessment of sequential stenoses and additional CCTA parameters [18].

## Conclusion

Personalised medicine and less invasive techniques are the present goal and expected future of healthcare. Preventing unnecessary invasive procedures, both diagnostic and therapeutic, is necessary to lower costs, complications and the burden of stable CAD on hospital resources. CT-FFR estimates lesion-induced ischaemia without additional (invasive) diagnostic testing and without changes to CCTA protocols. However, cost and reimbursement challenges currently prevent widespread availability.

Identification of high-risk individuals rather than risk factor-based subpopulations is crucial for adequate cardiovascular risk management. Plaque burden, plaque vulnerability and risk of CAD progression further guide treatment and risk modification. Future perspectives hold the promise of improved disease detection and characterisation, identification of new morphological features of prognostic value, inside and outside of the coronary arteries on CCTA with the use of AI, as attenuation-based CCTA images are especially suitable for automated assessment.

Further validation and selective adoption of the most promising, objective and reproducible techniques and algorithms will lead to maximal diagnostic value without overcomplicating patient management and guideline recommendations.
